# Physiological and Molecular Mechanisms of Nitrogen Regulation on Grain Quality in Cereal Crops at Later Stages

**DOI:** 10.3390/ijms27052125

**Published:** 2026-02-25

**Authors:** Aikui Guo, Hongfang Ren, Hongyan Yang, Zhihao Liang, Yuxing Li, Tingyu Dou, Yanling Ma, Huiquan Shen

**Affiliations:** 1 Jiangsu Coastal Area Institute of Agricultural Sciences, Yancheng 224002, China; gak16609326125@163.com (A.G.); yhy8373@163.com (H.Y.); nw88269663@163.com (Z.L.); 18702289708@163.com (Y.L.); 2 College of Forestry and Grassland, Nanjing Forestry University, Nanjing 210037, China; fangfang@njfu.edu.cn; 3 State Key Laboratory of Crop Gene Resources and Breeding, Institute of Crop Sciences, Chinese Academy of Agricultural Sciences, Beijing 100081, China; 15610416312@163.com; 4 Western Crop Genetic Alliance, Murdoch University, Perth, WA 6150, Australia

**Keywords:** late-stage nitrogen application, grain quality, physiological, molecular, nitrogen use efficiency, precision nitrogen management

## Abstract

Enhancing cereal grain quality while maintaining yield stability represents a pressing global challenge for sustainable agricultural development. Optimizing grain quality in cereal crops, which account for more than 60% of global dietary energy, relies heavily on managing nitrogen dynamics during the heading and grain-filling stages. Late-stage nitrogen application (from heading to early grain-filling stages) optimizes the temporal dynamics of nitrogen supply and exhibits substantial regulatory potential in mediating the yield–quality trade-off. Nitrogen availability can profoundly influence source–sink dynamics, carbon–nitrogen metabolic coordination, and the biosynthesis of storage reserves. This systematic review consolidates current understanding of the molecular and physiological mechanisms by which late-stage nitrogen application affects grain development and final quality in cereals, with a particular focus on major cereal crops including wheat, rice, and malting barley, which represent contrasting quality objectives and nitrogen management requirements. At the physiological level, late-stage nitrogen application delays functional leaf senescence, sustains photosynthetic carbon assimilation capacity, facilitates assimilate transport and partition to developing grains, and optimizes the accumulation dynamics and compositional profiles of starch and protein. At the molecular level, this review elucidates the sequential regulatory cascades governing nitrogen signal perception and transduction, the coordinated transcriptional networks underlying carbon–nitrogen metabolic crosstalk, and the expression dynamics of genes encoding starch biosynthetic enzymes and storage proteins. Integrating those recent research advances, this review also highlights several critical challenges currently facing the field. To address these challenges, we delineate promising avenues for future research including constructing time-series multi-omics frameworks, employing genome-editing technologies to functionally validate key regulatory genes and integrating artificial intelligence and big data analytics. The goal of this review is to establish a theoretical basis for precision nitrogen management strategies designed to optimize cereal crop production, targeting high yield, superior quality, and improved nitrogen use efficiency concurrently.

## 1. Introduction

Cereal grains serve not only as a primary staple food for human consumption but also as essential raw materials for the food processing, animal feed, and brewing industries. Major cereal crops, including wheat, rice, maize, and barley, collectively provide approximately 60% of global dietary energy and over 50% of protein intake [1]. The quality attributes of cereal grains directly determine the market value and end-use functionality of derived products. Consequently, achieving targeted regulation of grain quality while maintaining yield stability represents a pressing scientific challenge that warrants systematic investigation.

Grain quality is a multidimensional concept encompassing nutritional, functional, and processing attributes. Different cereal crops exhibit distinct quality requirements tailored to their specific end uses: bread wheat requires optimal glutenin composition for dough strength, cooking rice demands appropriate amylose content for eating quality, and malting barley necessitates strict protein content control (9.5–11.5%) for brewing performance [2,3,4]. These three crops were therefore selected as representative case systems in this review, as they encompass contrasting end-use demands and exemplify the diverse yield–quality trade-offs and nitrogen management strategies encountered in cereal production.

The grain-filling period is a decisive growth stage that establishes the key parameters for cereal grain quality [5]. The coordinated functioning of the ‘source–sink–flow’ system is fundamental to optimizing grain-filling efficiency. Functional leaves operate as the photosynthetic ‘source’ providing carbon assimilates, with the vascular bundles in stems and peduncles serving as the ‘flow’ pathway for assimilate translocation. Developing grains function as the ‘sink’ for storage compound accumulation [6,7]. The dynamic equilibrium between carbon and nitrogen metabolism during grain filling exerts decisive effects on grain quality attributes. Carbon metabolites are predominantly channeled into starch biosynthesis pathways, whereas nitrogen metabolites are primarily incorporated into storage proteins [8]. Complex interactions and metabolic competition between these two processes directly govern the starch-to-protein ratio in mature grains, thereby influencing final quality characteristics [9]. Understanding and manipulating the carbon–nitrogen metabolic balance during grain filling therefore represents a key breakthrough point for targeted grain quality improvement, with precision late-stage nitrogen management emerging as the principal agronomic approach to regulate this metabolic equilibrium.

Globally, agricultural nitrogen use efficiency (NUE) remains suboptimal, averaging only 40–50%, with substantial regional variation [10]. The prevailing “high-input and high-output” fertilization paradigm, particularly in intensive production regions, has imposed severe pressures on natural resources and environmental sustainability [11]. For instance, China’s annual nitrogen fertilizer consumption exceeds 30 million metric tons (expressed as pure nitrogen), accounting for over 30% of global usage, yet NUE remains at only 30–35%—substantially lower than the 50–60% achieved in many developed countries [12]. This inefficiency not only exhausts resources but also drives environmental degradation through nitrate leaching and nitrous oxide emissions [13].

In response to these challenges, nitrogen management strategies in cereal production are undergoing a fundamental shift from the traditional “high-input” paradigm toward precision-oriented approaches. Conventional fertilization practices, which rely on excessive and early-season nitrogen inputs to secure yield potential, often result in low nitrogen recovery efficiency, increased environmental losses, and limited flexibility for quality regulation. In contrast, precision nitrogen management emphasizes synchronizing nitrogen supply with crop demand in both temporal and spatial dimensions, aiming to maximize nitrogen use efficiency while minimizing environmental impacts. Within this emerging framework, optimizing nitrogen application timing has become a core regulatory lever, providing opportunities to fine-tune carbon–nitrogen metabolism during critical developmental windows rather than relying solely on increased fertilizer inputs [14].

Late-stage nitrogen application typically refers to topdressing nitrogen fertilizers during the period from heading to early grain filling in cereal crops. Accumulating evidence demonstrates that late-stage nitrogen application can significantly enhance NUE by supplementing nitrogen supply during the active grain-filling period, sustaining functional leaf activity, and extending the effective duration of grain filling [15]. Appropriately timed late-stage nitrogen application has been shown to substantially increase grain protein content, optimize amino acid profiles, and improve the physicochemical properties of starch [16]. Nevertheless, the effects of late-stage nitrogen application exhibit pronounced crop-specific variation, necessitating tailored management strategies. For bread wheat, where elevated protein content is a primary quality objective, the protein-enhancing effect of late-stage nitrogen constitutes its primary agronomic value. Conversely, for malting barley, which requires protein content within strictly defined limits, nitrogen management must ensure adequate grain filling without exceeding protein thresholds—a challenge that demands precise calibration of application timing, dosage, and nitrogen form within an inherently ‘narrow management window.’

Late-stage nitrogen application serves as a critical nexus linking nutrient management to grain quality formation; consequently, its physiological impacts and molecular regulatory mechanisms have been extensively characterized [17,18]. However, systematically elucidating the mechanistic basis by which late-stage nitrogen regulates grain quality and establishing scientifically grounded nitrogen management models remain significant challenges. Current knowledge gaps include: (i) incomplete understanding of the temporal dynamics of nitrogen signaling during grain filling; (ii) limited integration of physiological and molecular perspectives into unified regulatory frameworks; and (iii) insufficient translation of mechanistic insights into practical management guidelines across diverse genotype × environment scenarios.

Based on these considerations, this review is systematically organized into three focus areas: (i) effects of late-stage nitrogen application on grain quality attributes, (ii) physiological mechanisms governing source organ activity, assimilate transport, and sink development, and (iii) molecular regulatory networks controlling nitrogen signaling, carbon–nitrogen metabolic crosstalk, and storage compound biosynthesis. We further identify current research challenges and outline promising future directions, with the overarching aim of providing a robust theoretical foundation for developing precision nitrogen management strategies that simultaneously achieve high yield, superior quality, and enhanced resource-use efficiency in cereal crop production.

## 2. Effects of Late-Stage Nitrogen Application on Grain Quality Attributes

**Figure 1:**
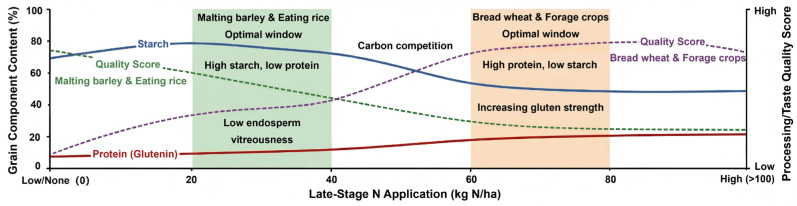
Schematic diagram of the effects of late-stage nitrogen application on grain starch and protein accumulation.

Late-stage nitrogen application differentially modulates the accumulation of protein and starch in cereal grains through regulation of carbon–nitrogen metabolic balance (Figure 1). These quality trait modifications exhibit pronounced crop-specific patterns that directly determine grain processing suitability and end-use value.

### 2.1. Modulation of Protein Content and Composition

#### 2.1.1. Dose–Response Patterns of Protein Accumulation

In response to late-stage nitrogen application, the grain protein content follows a characteristic dose–response pattern, wherein protein content does not increase linearly with nitrogen input but rather exhibits a saturation effect beyond a critical threshold [15]. This nonlinear response reflects the physiological constraints on nitrogen assimilation and storage protein biosynthesis capacity in developing grains. Importantly, late-stage nitrogen application influences not only total protein content but also exerts significant regulatory effects on storage protein composition, which ultimately determines processing quality attributes [14].

#### 2.1.2. Wheat Storage Proteins and Bread-Making Quality

Cereal grain storage proteins are primarily categorized into two principal classes: prolamins (gliadins in wheat and hordeins in barley) and glutelins (glutenins), with their relative proportions critically influencing end-use quality [19]. In wheat grains, gliadins confer dough extensibility, whereas glutenins determine dough elasticity and strength [20]. The ratio between these protein fractions, particularly the glutenin-to-gliadin ratio, is a key determinant of bread-making quality.

Late-stage nitrogen application preferentially promotes the accumulation of high-molecular-weight glutenin subunits (HMW-GSs), thereby enhancing gluten strength and improving bread-baking performance [21]. Research has found that nitrogen topdressing after anthesis significantly increased the glutenin-to-gliadin ratio in wheat grains, resulting in prolonged dough stability time and improved loaf volume [22].

#### 2.1.3. Rice Storage Proteins and Eating Quality

In rice, glutelins constitute the predominant storage protein fraction, accounting for approximately 80% of total protein, while prolamins represent a relatively minor component. Late-stage nitrogen application can promote balanced accumulation of glutelin subunits, potentially improving the nutritional quality of rice grain [23]. However, optimizing the trade-off between nitrogen fertilization rate and desirable eating quality parameters in rice production requires careful management to avoid adverse effects on grain quality. Excessive nitrogen application may lead to disproportionate increases in prolamin components relative to glutelins, adversely affecting the cooking and eating quality by increasing grain hardness and reducing the characteristic stickiness of cooked rice [24].

#### 2.1.4. Barley Hordeins and Malting Quality

For malting barley, the relationship between nitrogen supply and protein composition assumes particular significance due to the stringent quality requirements of the brewing industry. The Kolbach index—the ratio of soluble to total nitrogen—is a definitive indicator of malt modification, where a range of 38–45% signifies optimal proteolytic degradation during the malting process [25].

The effect of late-stage nitrogen application on the Kolbach index exhibits a complex nonlinear pattern. Moderate nitrogen application can increase the proportion of soluble proteins and improve malt extract yield [26], while excessive nitrogen application promotes the over-accumulation of insoluble proteins—specifically D-hordein—thereby suppressing the Kolbach index and compromising overall malt quality [27]. Among the different hordein fractions, D-hordein exhibits the highest sensitivity to nitrogen supply, with its accumulation responding disproportionately to increased nitrogen availability [28]. Elevated D-hordein content is closely associated with increased proportions of “vitreous endosperm,” which resists hydration and enzymatic modification during malting, severely compromising brewing efficiency and final beer quality [29].

#### 2.1.5. Amino Acid Composition and the Dilution Effect

Late-stage nitrogen application, while increasing total protein content, simultaneously affects the amino acid composition pattern of grain proteins [30]. A well-documented phenomenon termed the “dilution effect” describes the inverse relationship frequently observed between total protein content and the relative proportions of essential amino acids, particularly lysine and tryptophan [31]. This effect occurs because late-stage nitrogen application preferentially stimulates the synthesis of storage proteins (prolamins and glutelins), which are characteristically deficient in essential amino acids, thereby shifting the overall amino acid profile towards enrichment in non-essential amino acids such as glutamine and proline [9,32]. Nevertheless, emerging evidence suggests that strategic optimization of nitrogen application timing and form may partially mitigate the dilution effect, enabling simultaneous increases in protein content while maintaining a relatively balanced amino acid profile [33].

### 2.2. Regulation of Starch Accumulation and Physicochemical Properties

#### 2.2.1. Carbon–Nitrogen Competition and Starch Accumulation

Starch constitutes the predominant storage compound in cereal grains, accounting for approximately 60–75% of grain dry weight [34]. The effect of late-stage nitrogen application on starch accumulation characteristically exhibits a competitive “trade-off” relationship with its effect on protein biosynthesis [35]. This carbon–nitrogen competition stems from two interconnected mechanisms: firstly, amino acid biosynthesis diverts carbon skeletons (particularly 2-oxoglutarate and oxaloacetate) from starch synthesis; secondly, the substantial requirements for ATP and reducing equivalents in protein biosynthesis compete with starch biosynthetic pathways for shared metabolic resources [8,36].

In terms of conflicting evidence and sources of heterogeneity, reports on how late-stage nitrogen application affects starch accumulation are not fully consistent, with studies showing decreases, negligible changes, or even slight increases in starch content. These discrepancies likely arise from (i) baseline nitrogen status and the marginal response to additional N, (ii) timing relative to the starch deposition window (e.g., 0–10 vs. 10–20 DAA), (iii) genotype-specific sink capacity and nitrogen remobilization efficiency, and (iv) concurrent environmental constraints (radiation/temperature/water) that determine whether carbon supply becomes limiting [37,38,39]. Thus, whether late-stage N reduces starch primarily depends on whether the treatment shifts the system toward carbon limitation (protein is favored and starch is diluted) or enhances source capacity and grain-filling duration (starch is maintained).

#### 2.2.2. Enzymatic Regulation of Starch Biosynthesis

Grain starch biosynthesis is accomplished through the coordinated action of a suite of enzymes, with ADP-glucose pyrophosphorylase (AGPase) widely recognized as the rate-limiting enzyme controlling carbon flux into starch [40]. Adequate nitrogen supply can maintain a high level of AGPase activity, ensuring sufficient substrate availability for starch synthesis [41]. However, excessive nitrogen application may paradoxically suppress starch accumulation by inhibiting AGPase activity or downregulating its gene expression, illustrating the importance of optimal nitrogen dosing. It should be noted that the thresholds defining “adequate” versus “excessive” nitrogen supply are inherently crop-, cultivar-, and environment-specific, and therefore should be interpreted within specific agroecological contexts rather than as fixed universal values [42]. As the downstream products of AGPase, the relative activities of soluble starch synthase (SSS) and granule-bound starch synthase (GBSS) determine the proportional synthesis of amylopectin and amylose, respectively [43,44]. Starch-branching enzymes (SBEs) and debranching enzymes (DBEs) further modulate the fine structure of amylopectin by creating and trimming branch points [45,46]. Nitrogen availability has been shown to regulate the relative activities of these enzymes, thereby influencing the crystalline architecture, granule morphology, and physicochemical properties of starch [47]. Late-stage nitrogen application may induce subtle shifts in the amylose-to-amylopectin ratio, although the direction and magnitude of this effect vary considerably among crop species and varieties [48].

#### 2.2.3. Pasting Properties and Processing Quality

Late-stage nitrogen application influences starch pasting properties—including peak viscosity, breakdown, setback, and gelatinization temperature—through its effects on both starch content and molecular composition [49,50]. Elevated nitrogen supply typically results in decreased peak viscosity and increased gelatinization temperature, changes that carry significant implications for processing quality. These alterations are closely linked to starch–protein matrix interactions: higher protein content may physically restrict the swelling of starch granules during heating, thereby modifying the gelatinization process and final product texture [51]. For rice, starch gelatinization characteristics directly determine the texture of cooked rice, so late-stage nitrogen application needs to strike a balance between protein nutrition and eating quality [52]. In malting barley, late-stage nitrogen application may alter the size distribution of starch granules by affecting the balance between carbon and nitrogen metabolism during grain development [53]. Excessive nitrogen supply promotes thickening of the protein matrix surrounding starch granules, which may increase the proportion of small B-type granules at the expense of larger A-type granules—a shift that is detrimental to malt dissolution and extract yield [54].

β-amylase, a key enzyme in the malting and mashing process responsible for cleaving maltose units from starch chains, is indirectly influenced by the protein metabolic status of the grain [55]. Moderate late-stage nitrogen application can maintain relatively high β-amylase activity, supporting efficient starch hydrolysis during mashing. In contrast, excessive nitrogen application exerts an inhibitory effect on β-amylase activity, potentially through mechanisms involving altered protein–enzyme interactions or modified grain ultrastructure [56]. These findings underscore the importance of precise nitrogen management for optimizing both starch quantity and functionality in malting barley.

In terms of why rheological responses can diverge, conflicting trends in pasting/rheological parameters (e.g., peak viscosity, breakdown, and setback) are expected because these traits integrate multiple coupled determinants rather than starch content alone. Late-stage N simultaneously alters protein accumulation, starch structure (amylose/amylopectin features), and non-starch polysaccharides, thereby shifting starch swelling and granule disintegration in opposite directions depending on genotype and environment [57,58,59]. Consequently, similar increases in grain protein can translate into different viscosity profiles when starch architecture or cell-wall composition differs, highlighting the need to interpret rheological outcomes in a composition–structure framework instead of a single-factor (N dose) framework.

### 2.3. Influence on Free Amino Acids and Functional Components

#### 2.3.1. Free Amino Acids and Their Significance

Although the content of Free Amino Acids (FAAs) in mature grains is substantially lower than that of protein-bound amino acids, FAAs play important roles in seed vitality, germination metabolism, and the quality of downstream processed products [60]. Late-stage nitrogen application can significantly increase total FAA content in grains, with particularly pronounced effects on amino acids central to nitrogen metabolism, including glutamic acid, aspartic acid, and their amide derivatives glutamine and asparagine [61].

For malting barley and the brewing industry, Free Amino Nitrogen (FAN) in malt wort provides the essential nitrogen source for yeast metabolism during fermentation. The brewing industry typically specifies a minimum FAN level of >130 mg/L to ensure adequate yeast nutrition and fermentation performance [62]. Late-stage nitrogen application can effectively increase malt FAN levels, thereby improving fermentation vigor and consistency [63]. Different amino acids contribute differentially to beer flavor profiles, and excessive accumulation of certain amino acids—particularly branched-chain amino acids (leucine, isoleucine, and valine) and sulfur-containing amino acids—may lead to the production of undesirable flavor compounds during fermentation, including fusel alcohols and sulfur off-flavors [64]. Therefore, optimal nitrogen management for brewing barley must consider not only total FAN content but also the amino acid composition profile.

#### 2.3.2. β-Glucan Content and Its Dual Significance

β-Glucan, a mixed-linkage (1 → 3, 1 → 4)-β-D-glucan, is a major structural component of barley grain cell walls, with content typically ranging from 3% to 7% of grain dry weight [65]. This polysaccharide has attracted considerable interest for its demonstrated health benefits, including cholesterol reduction, glycemic control, and promotion of beneficial gut microbiota [66]. From a nutritional perspective, elevated β-glucan content is therefore desirable.

However, from a brewing perspective, elevated β-glucan levels (>4.5%) pose substantial processing difficulties. Inadequately degraded β-glucan increases wort viscosity, severely impairs lautering (wort filtration) efficiency, and may cause haze formation in the final beer product [67]. The effect of late-stage nitrogen application on β-glucan content remains controversial. Some studies suggest that elevated nitrogen supply may influence β-glucan accumulation by modulating cell-wall biosynthetic pathways, but the magnitude and direction of this effect appear to be highly dependent on genotypes or environmental conditions [68]. Resolving this uncertainty is a critical research priority for optimizing nitrogen management in barley production across diverse end-use markets.

With regard to conflicting reports and a reconciliatory view, evidence regarding the effect of late-stage N on β-glucan is mixed. This is plausible because β-glucan content reflects the balance between (i) cell-wall deposition/maintenance and (ii) endosperm starch accumulation that can dilute cell-wall polysaccharides [69]. Late-stage N that primarily increases grain protein and prolongs canopy greenness under limited carbon supply may favor higher relative β-glucan and higher wort viscosity risk, whereas N that enhances grain filling and starch deposition under high radiation can lead to unchanged or diluted β-glucan [70,71]. Therefore, β-glucan responses should be interpreted jointly with carbon supply conditions and end-use targets (health vs. brewing).

## 3. Physiological Mechanisms of Late-Stage Nitrogen Application Regulating Grain Filling and Quality Formation

### 3.1. Maintaining Photosynthetic Capacity of Source Organs

#### 3.1.1. Leaf Senescence and Nitrogen-Mediated Delay

Leaf senescence is a genetically programmed developmental process characterized by chlorophyll degradation, progressive decline in photosynthetic capacity, and systematic protein hydrolysis for nitrogen remobilization to developing reproductive sinks [72]. In wheat, flag leaves typically exhibit visible signs of senescence at 10–15 days after anthesis (DAA), with chlorophyll content declining at a rate of approximately 1–2% per day thereafter [73]. This progressive loss of photosynthetic capacity directly limits the carbon supply available for grain filling during the critical period of storage compound accumulation. Adequate nitrogen availability during this critical period can effectively suppress the expression of senescence-associated genes (SAGs), thereby delaying the onset and progression of leaf senescence [74]. The molecular mechanisms underlying this effect involve nitrogen-mediated modulation of hormonal balance, particularly the cytokinin-to-ethylene ratio and direct effects on chloroplast protein turnover rates. Empirical studies have demonstrated that moderate nitrogen application at the heading stage extends the functional longevity of wheat flag leaves by 5–10 days and that of rice flag leaves by 7–12 days, providing an extended window for photosynthetic carbon assimilation [75,76]. Importantly, this delayed senescence effect is most beneficial under late-stage nitrogen application because the grain-filling window is developmentally constrained; thus, extending functional leaf duration during 0–20 DAA has a disproportionately large impact on nitrogen remobilization efficiency and final grain protein accumulation compared with earlier vegetative stages.

#### 3.1.2. Protection of Photosynthetic Apparatus

Late-stage nitrogen application exerts pronounced protective effects on the photosynthetic apparatus through multiple interconnected mechanisms. Firstly, it delays the degradation of Photosystem II (PSII) reaction centers, which serve as the initial site of photosynthetic electron transport. Within PSII, the D1 protein undergoes continuous photodamage under illumination and requires constant repair and turnover to maintain functional integrity [77]. Under conditions of sufficient nitrogen supply, the turnover rate of D1 protein is sustained through continued de novo synthesis, enabling the maximum quantum efficiency of PSII (Fv/Fm) to remain at relatively high levels throughout the grain-filling period. Zhou et al. [78] reported that rice plants receiving late-stage nitrogen application exhibited 8–12% higher Fv/Fm values during the mid-to-late grain-filling stage compared with untreated controls, indicating enhanced PSII functionality and sustained photosynthetic electron transport capacity. This maintenance of PSII efficiency directly supports continued adenosine triphosphate (ATP)and nicotinamide adenine dinucleotide phosphate (NADPH) generation for carbon fixation during the critical period of grain filling. Secondly, late-stage nitrogen application helps maintain both the activity and abundance of Ribulose-1,5-bisphosphate Carboxylase/Oxygenase (RuBisCO). As the rate-limiting enzyme catalyzing photosynthetic carbon assimilation, RuBisCO constitutes approximately 50% of the total soluble protein in C_3_ leaves, serving as the primary reservoir for foliar nitrogen [79]. During senescence, RuBisCO is preferentially targeted for proteolytic degradation to release nitrogen for remobilization to developing grains—a process that inevitably compromises photosynthetic capacity. Late-stage nitrogen application alleviates this degradation pressure by providing an exogenous nitrogen source, thereby reducing the imperative for internal nitrogen recycling and extending the functional duration of RuBisCO activity. The magnitude of this effect has been quantified in several studies. Lawlor [80] demonstrated that nitrogen application at the heading stage maintained wheat flag leaf RuBisCO activity at over 70% of its peak value at 20 DAA, whereas untreated controls retained only approximately 50% of peak activity. This sustained RuBisCO activity translates directly into prolonged carbon assimilation capacity and enhanced assimilate supply to developing grains, ultimately supporting greater accumulation of storage compounds. Collectively, maintaining PSII efficiency and RuBisCO capacity provides a physiological basis for how late-stage nitrogen application sustains carbon assimilation and supports continued storage compound accumulation during grain filling.

#### 3.1.3. Interspecific Variation in Nitrogen Response

The response of cereal crops to late-stage nitrogen application exhibits considerable interspecific variation, reflecting inherent differences in flag leaf longevity, developmental dynamics, and quality objectives.

Wheat flag leaves typically maintain functionality for 35–45 days post-anthesis, providing a moderately flexible window for late nitrogen management interventions [14]. Rice flag leaves exhibit a somewhat longer functional period of 40–50 days, offering even greater flexibility for nitrogen application timing [81]. The extended functional duration in rice may be attributed to its adaptation to high-radiation tropical and subtropical environments, where sustained photosynthetic capacity provides competitive advantages. In contrast, barley flag leaves have a comparatively shorter functional period of only 25–35 days, rendering the timing of late-stage nitrogen application particularly critical [8]. This compressed developmental timeline reflects the determinate growth habit and rapid grain-filling characteristic of barley, which evolved as an early-maturing crop adapted to environments with terminal drought or heat stress. Hollmann et al. [82] and Singh [83] reported that nitrogen application at 7–10 days after heading in barley effectively maintained elevated chlorophyll content in flag leaves until the late grain-filling stage, and extended functional leaf duration by 3–7 days. This interspecific contrast implies that “late-stage” nitrogen has a narrower effective timing window in barley than in wheat or rice, thereby increasing the likelihood of variable outcomes across experiments when timing is not precisely comparable.

#### 3.1.4. Special Considerations for Malting Barley

For malting barley, the management of leaf senescence and the associated “stay-green” phenotype must be carefully balanced against grain protein content requirements. Excessively prolonged leaf functionality, while beneficial for carbon assimilation, may result in continued nitrogen translocation to grains and consequent elevation of grain protein content beyond the acceptable threshold of 9.5–11.5% required by the malting industry [27,84].

This intrinsic trade-off between maximizing photosynthetic duration and constraining protein accumulation exemplifies the “narrow management window” challenge specific to malting barley production. Unlike bread wheat or rice, where extended nitrogen supply is generally beneficial for quality and moderate nitrogen management suffices, malting barley requires precise calibration of nitrogen application timing and rate to achieve the dual objectives of adequate grain filling and controlled protein accumulation. This unique challenge underscores the need for crop-specific nitrogen management guidelines and diagnostic tools tailored to quality-oriented production systems. Accordingly, the same physiological “stay-green” benefit can be desirable in wheat but risky in malting barley, which partially explains inconsistent quality responses among cereals.

### 3.2. Enhancing Assimilate Transport and Sink Establishment

#### 3.2.1. Regulation of Sucrose Metabolism Enzymes

The transport of photoassimilates from source to sink organs fundamentally relies on efficient phloem loading and unloading processes, which are mediated by a coordinated network of metabolic enzymes [85].

Sucrose, serving as the predominant transport carbohydrate in cereal crops, undergoes a series of metabolic transformations regulated by multiple key enzymes that collectively determine the efficiency of carbon flux from leaves to grains. Sucrose Phosphate Synthase (SPS) catalyzes sucrose biosynthesis in source leaves and functions as a critical regulatory enzyme controlling the export of photosynthetic products [86]. The activity of SPS is positively correlated with sucrose export rate and represents a potential target for improving assimilate supply to developing grains. Conversely, Sucrose Synthase (SuSy) predominantly operates in sink organs, where it catalyzes the reversible cleavage of sucrose to provide UDP-glucose and fructose as substrates for starch biosynthesis and cellular metabolism [87]. Late-stage nitrogen application significantly enhances the activities of these sucrose-metabolizing enzymes. Deng et al. [88] demonstrated that nitrogen application at the heading stage increased SPS activity in rice leaves by 15–25% while simultaneously sustaining SuSy activity in developing grains. This coordinated enhancement effectively increases sucrose export capacity from source leaves while accelerating the conversion of imported sucrose into starch precursors in sink tissues.

Additionally, Cell-Wall Invertase (CWIN) plays a pivotal role in apoplastic sucrose unloading at the sink interface. Located in the cell-wall space of sink tissues, CWIN irreversibly hydrolyzes sucrose into glucose and fructose, creating a concentration gradient that drives continued sucrose import. The activity of CWIN is positively modulated by nitrogen availability, providing another mechanism through which late-stage nitrogen application enhances sink strength [89]. Thus, late-stage nitrogen can simultaneously reinforce source export (SPS) and sink utilization/unloading (SuSy/CWIN), a coordinated response that is most relevant during the grain-filling window.

#### 3.2.2. Maintenance of Phloem Transport Function

As the long-distance conduit for assimilate translocation, the structural integrity and functional activity of the phloem directly determine the efficiency of source-to-sink carbon exchange [90].

Late-stage nitrogen application contributes to maintaining phloem transport capacity, sustaining phloem parenchyma cell vitality, delaying senescence of the sieve element–companion cell complex, and preserving the functional integrity of plasmodesmatal connections that facilitate symplastic transport [54].

Isotopic tracer studies have provided direct evidence for enhanced assimilate translocation following late-stage nitrogen application. Masclaux-Daubresse et al. [91] reported that in wheat plants receiving late-stage nitrogen treatment, the transfer efficiency of ^14^C-labeled photoassimilates from flag leaves to developing grains increased by 18–22% compared with control plants. Concurrently, the remobilization efficiency of non-structural carbohydrates (NSCs) stored in stem and leaf sheath tissues was also significantly improved in nitrogen-treated plants [92]. This finding indicates that late-stage nitrogen application not only promotes the transport of currently synthesized photosynthates but also enhances the redistribution capacity of pre-anthesis carbon reserves. Furthermore, nitrogen supply during the critical period from spike differentiation to early grain filling can promote vascular bundle development in the peduncle and rachis. Ren et al. [93] demonstrated that moderate nitrogen application during this window increased phloem cross-sectional area and sieve tube density, thereby enhancing the physical capacity of the vascular system to deliver assimilates to developing grains. Together, these data support the idea that late-stage nitrogen can improve both “real-time flux” and “reserve remobilization,” helping to explain yield/quality gains when carbon supply is not limiting.

#### 3.2.3. Optimization of Grain-Filling Dynamics

The grain-filling process can be quantitatively characterized by three key parameters: filling onset time, mean filling rate, and filling duration.

Wang et al. [94] reported that nitrogen application at the wheat heading stage prolonged grain-filling duration by 4–7 days, resulting in a corresponding increase in thousand-grain weight of 5–10%. Similarly, Zhou et al. [95] demonstrated that late-stage nitrogen application in rice primarily enhances grain weight through extending the active filling duration rather than by increasing the maximum filling rate. This pattern suggests that the primary benefit of late nitrogen supply lies in preventing premature termination of grain filling rather than accelerating the filling process per se. However, excessive nitrogen supply may induce excessive vegetative growth, delay canopy senescence beyond the optimal timing, and ultimately postpone physiological maturity [96]. Therefore, optimizing late-stage nitrogen management requires careful calibration to maximize beneficial effects on grain filling while avoiding the detrimental consequences of over-application. This nonlinear response provides a physiological basis for the conflicting reports in the literature, where differences in late-N dose, timing, and carbon supply conditions can shift outcomes from beneficial to inhibitory.

#### 3.2.4. Crop-Specific Considerations in Sink Establishment

The anatomical characteristics of grain structure impose crop-specific constraints on assimilate unloading efficiency. In barley, the caryopsis is tightly enclosed by the adherent husk (lemma and palea), making imported assimilates traverse additional cell layers before reaching the developing endosperm [97].

The functional state of endosperm transfer cells (ETCs) is particularly critical for efficient assimilate unloading. These specialized cells, located at the maternal–filial interface, are characterized by extensive cell-wall ingrowths that substantially amplify the plasma membrane surface area available for active nutrient transport [98]. Late-stage nitrogen application has been shown to maintain elevated metabolic activity in ETCs, thereby promoting efficient sucrose transfer from maternal tissues to the developing endosperm [99]. This enhanced transfer cell functionality may partially compensate for the anatomical constraints imposed by the adherent husk structure in barley. In barley, this anatomical constraint may increase the sensitivity of grain filling to the synchrony between nitrogen availability and carbon flux, contributing to greater variability in late-N effects across studies.

### 3.3. Modulating Carbon–Nitrogen Partitioning and Allocation

#### 3.3.1. Metabolic Basis of Carbon–Nitrogen Trade-Off

**Figure 2:**
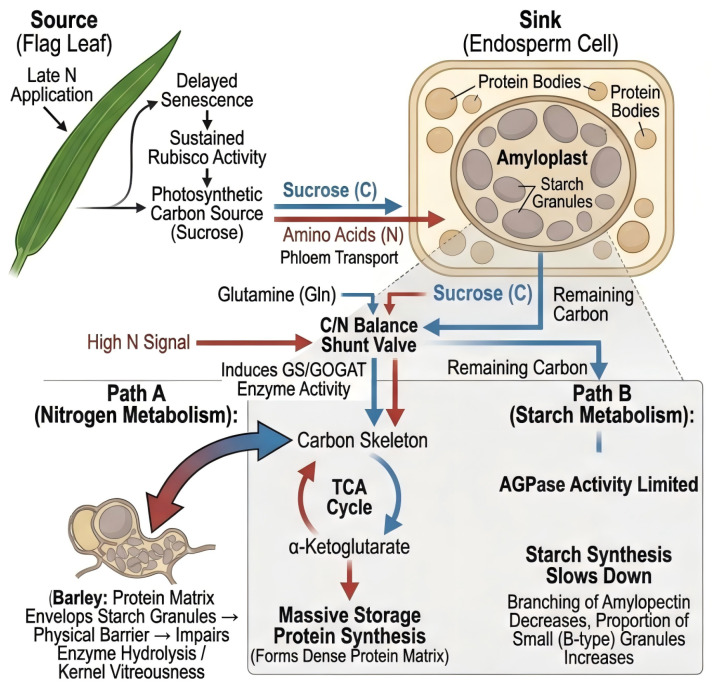
Conceptual framework of source–sink metabolic flux dynamics in cereal crops under late-stage nitrogen application. N, nitrogen; C, carbon; TCA, tricarboxylic acid; GS, glutamine synthetase; GOGAT, glutamate synthase.

Late-stage nitrogen application directly influences the carbon-to-nitrogen ratio in developing grains by altering the metabolic flux distribution between carbon and nitrogen assimilation pathways [22] (Figure 2).

With increased nitrogen input during the grain-filling period, the supply of amino acids to developing grains is enhanced, leading to elevated storage protein biosynthesis.

Concurrently, a portion of the carbon skeleton pool is diverted from carbohydrate metabolism toward amino acid synthesis, as 2-oxoglutarate and other organic acids are withdrawn from the tricarboxylic acid (TCA) cycle for nitrogen assimilation via the glutamine synthetase/glutamate synthase (GS/GOGAT) pathway [100]. This diversion reduces the substrate availability for starch biosynthesis, establishing a direct metabolic competition between protein and starch accumulation. The substantial energetic demand of protein synthesis in terms of ATP and NADPH consumption further competes for metabolic resources, thereby limiting the carbon flux directed toward starch synthesis [35]. Accordingly, the net effect of late-stage nitrogen on starch depends on whether carbon supply (photosynthesis + reserve mobilization) is sufficient to match the elevated nitrogen-driven anabolic demand.

#### 3.3.2. Enzymatic Regulation of Carbon–Nitrogen Balance

Elevated nitrogen supply stimulates the activities of GS and GOGAT, which catalyze the primary assimilation of ammonium into amino acids [101].

Simultaneously, nitrogen status influences the activities of starch biosynthetic enzymes, including AGPase, starch synthases (SSs), and SBE [40,44,46].

Evidence suggests that moderate late-stage nitrogen application can maintain balanced activities of both enzyme systems, supporting concurrent accumulation of starch and protein within acceptable ranges for specific quality objectives. However, excessive nitrogen supply may tip the balance toward nitrogen metabolism at the expense of starch accumulation [102]. This enzymatic crosstalk suggests that precision nitrogen management—rather than simple maximization or minimization of nitrogen supply—is required to achieve optimal grain composition. This enzyme-level reciprocity offers a mechanistic interpretation for why similar late-N treatments can increase protein consistently while starch responses vary among environments and genotypes.

#### 3.3.3. Crop-Specific Requirements for Carbon–Nitrogen Balance

Different cereal crops exhibit vastly different requirements for carbon–nitrogen balance regulation, reflecting their distinct quality objectives and end-use specifications.

Bread wheat is typically targeted for high protein content (>12%) to ensure adequate gluten strength for bread-making applications [103]. Studies have demonstrated that nitrogen application at the heading to early grain-filling stages can increase wheat grain protein content by 1–3%, with corresponding improvements in gluten strength indices such as the sedimentation value and farinograph stability time [14].

*Japonica* rice requires moderate protein content (typically 7–9%) to maintain optimal eating and cooking quality. Unlike wheat, excessive protein accumulation in rice grains negatively affects palatability by increasing grain hardness and reducing the characteristic stickiness that is prized in *Japonica* rice consumption [104]. The optimal strategy for *Japonica* rice involves moderate nitrogen application at heading combined with controlled nitrogen supply during early grain filling [105]. For example, studies on Japonica rice in the Yangtze River region have reported representative late-stage application ranges of approximately 30–60 kg N ha^−1^ (typically as urea), although these values vary considerably depending on soil fertility, cultivar characteristics, and baseline nitrogen management [106].

Malting barley represents the most challenging scenario due to the stringent and bidirectional constraints on grain composition. The malting industry requires grain protein content within a narrow optimal range of 9.5–11.5%, while simultaneously demanding high starch content (>60%) [107]. Protein content below 9.5% may result in insufficient enzymatic activity during malting. Conversely, protein content exceeding 11.5% leads to poor malt quality characterized by excessive protein modification and reduced fermentable extract [3]. This dual constraint means that late-stage nitrogen management in malting barley must navigate an extremely narrow window, demanding precise integration of application timing, dosage, and nitrogen form. Therefore, late-stage nitrogen management in malting barley must navigate an exceptionally narrow window between sustaining source function and restricting excessive grain protein accumulation, demanding precise integration of timing, dosage, and nitrogen form.

Overall, late-stage nitrogen application improves grain filling and quality by sustaining source activity, strengthening assimilate transport, and modulating carbon–nitrogen allocation. To explain how these physiological responses are initiated and coordinated, Section 4 summarizes the underlying molecular regulatory mechanisms.

## 4. Molecular Mechanisms of Late-Stage Nitrogen Application Regulating Grain Quality

**Figure 3:**
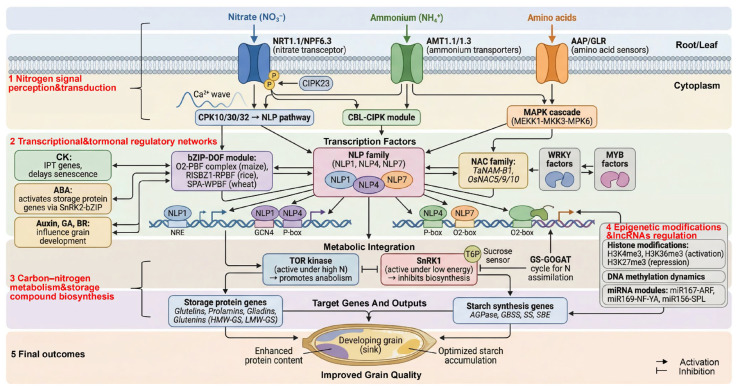
A schematic model illustrating the molecular mechanisms underlying grain quality regulation by late-stage nitrogen (LN) application in cereals. ABA, abscisic acid; AGPase, ADP-glucose pyrophosphorylase; AMT, ammonium transporter; BRs, brassinosteroids; CBL, calcineurin B-like protein; CIPKs, CBL-interacting protein kinases; CK, cytokinin; CPKs, calcium-sensor protein kinases; DOF, DNA-binding with One Finger; GA, gibberellin; GBSS, granule-bound starch synthase; GLR, glutamate receptor-like; GS, glutamine synthetase; GOGAT, glutamate synthase; HMW-GSs, high-molecular-weight glutenin subunits; LMW-GSs, low-molecular-weight glutenin subunits; MAPK, mitogen-activated protein kinase; N, nitrogen; NLP, NIN-like protein; NPF, peptide transporter family; NRE, nitrate-responsive cis-element; NRT, nitrate transporter; NUE, nitrogen use efficiency; SBE, starch-branching enzyme; SnRK1, sucrose non-fermenting 1-related kinase 1; SS, starch synthases; T6P, trehalose-6-phosphate; TOR, target of rapamycin.

The molecular basis for grain quality regulation by late-stage nitrogen application encompasses four interconnected regulatory layers: (i) nitrogen signal perception and transduction, (ii) remodeling of transcriptional and hormonal regulatory networks, (iii) coordinated expression of genes governing carbon–nitrogen metabolism and storage compound biosynthesis, and (iv) fine-tuning through epigenetic modifications and non-coding RNAs (Figure 3).

### 4.1. Nitrogen Signal Perception and Transduction During Grain Filling

#### 4.1.1. Nitrogen-Sensing Systems and Tissue Specificity

Nitrate sensing: Nitrate perception is primarily mediated by NRT1/NPF (nitrate transporter 1/peptide transporter family) [108]. NRT1.1 (NPF6.3) functions as a dual-affinity transceptor whose phosphorylation status, mediated by calcineurin B-like protein (CBL)-interacting protein kinases 23 (CIPK23), determines the affinity switch between high and low modes [109,110]. Of particular relevance to late-stage nitrogen management, natural variation in rice *OsNRT1.1B* explains NUE differences between Japonica and Indica subspecies and likely underpins differential responsiveness to post-heading nitrogen [111]. The wheat *TaNRT2.1*–*TaNAR2.1* complex mediates high-affinity nitrate uptake, with expression during grain filling positively correlated with late nitrogen responsiveness [112]. *OsNRT2.3a* is specifically expressed in vascular tissues, determining long-distance nitrate transport, and may serve as a bottleneck for nitrogen delivery to developing panicles following late application [113]. Of particular relevance to late-stage nitrogen application, vascular/tissue-specific NRT expression can influence how efficiently exogenous nitrogen is delivered to developing spikes/panicles during grain filling, thereby shaping grain protein outcomes.

Ammonium sensing: The ammonium transporter (AMT) family mediates transmembrane ammonium uptake, with certain members (*AMT1;1* and *AMT1;3*) exhibiting transceptor characteristics through C-terminal cytoplasmic domain interactions with Protein Phosphatase 2C (PP2C) phosphatases [114,115]. In rice, *OsAMT1;1* and *OsAMT1;2* mediate ammonium uptake and radial transport, respectively, with their activities regulated by glutamine levels and phosphorylation status [116,117]. In maize, *ZmAMT1.1a* and *ZmAMT1.3* exhibit functional differentiation between high-affinity uptake and signal regulation [118,119].

Organic nitrogen sensing: Amino acid signals are perceived through glutamate receptor-like (GLR) and Amino Acid Permease (AAP) transporters/receptors [120,121]. *OsGLR* and *OsAAP* members are highly expressed in developing endosperm, participating in amino acid sensing and storage protein synthesis regulation [122,123].

During grain filling, nitrogen sensing exhibits pronounced tissue specificity that is central to the effects of late-stage nitrogen application. In source organs (flag leaves), NRT/NPF, AMT, and AAP primarily govern senescence and nitrogen remobilization [124]. In sink tissues (rachis, glumes, and grains), NPF/GLR/AAP directly influence nitrogen allocation to developing grains [125,126]. The balance of sensor expression between source and sink tissues thus determines whether exogenous nitrogen supplied at late stages is preferentially used to sustain source photosynthetic capacity or channeled directly into grain protein accumulation—a distinction with critical implications for the quality outcomes of different cereal crops.

#### 4.1.2. Signal Transduction Relevant to Late-Stage Nitrogen Responses

Nitrogen signals are transmitted to the nucleus through well-characterized Ca^2+^ signaling, protein kinase cascades, and post-translational modification pathways [127]. Rather than recapitulating these general mechanisms, we focus here on the components with demonstrated relevance to post-anthesis nitrogen responses. During grain filling, these signaling modules are primarily interpreted in source–sink contexts (flag leaf senescence, nitrogen remobilization, and endosperm storage deposition), but this differs from their roles in regulating root foraging and vegetative biomass accumulation.

The CPK10/30/32–NLP7 module is of particular significance because it enables rapid membrane-to-nucleus signal transduction within minutes of nitrate perception [128,129], providing a molecular explanation for the swift transcriptional responses observed after late-stage nitrogen application. This rapid signaling is critical during grain filling, when the developmental window for influencing storage compound accumulation is inherently limited [130,131,132].

The duration and intensity of nitrogen signaling are fine-tuned by E3 ubiquitin ligases, such as Nitrogen Limitation Adaptation (NLA) and Arabidopsis Tóxicos en Levadura 31 (ATL31), and regulatory 14-3-3 proteins [133,134]. These components control the stability and activity of key nitrogen regulatory proteins including NRT, nitrate reductase (NR), and GS [135,136]. Importantly, the activity of NLA determines whether exogenous nitrogen is efficiently channeled toward grain nitrogen supply or is attenuated through proteasomal degradation of nitrogen transporters—a regulatory checkpoint that may influence the effectiveness of late-stage nitrogen application across different genotypes.

### 4.2. Nitrogen-Responsive Transcriptional Regulatory Network

#### 4.2.1. NLP Transcription Factors as Master Regulators

The NIN-like protein (NLP) family has emerged as the primary transcription factor family mediating plant responses to nitrate signals [137]. NLPs recognize and bind to nitrate-responsive cis-elements (NREs) in target gene promoters and undergo rapid nuclear translocation upon nitrate exposure [138].

In rice, *OsNLP1* and *OsNLP4* directly activate the expression of genes encoding nitrogen transporters and GS, and their overexpression significantly improves nitrogen use efficiency [139,140]. Critically for the context of late-stage nitrogen management, the NLP regulatory module maintains nitrogen uptake and redistribution capacity within the root–leaf–panicle system during grain filling, ensuring sufficient amino acid precursor supply for storage protein biosynthesis [141]. This function makes NLPs the primary molecular targets through which late nitrogen application exerts its effects on grain protein content.

#### 4.2.2. bZIP and DOF Factors Linking Nitrogen to Storage Protein Genes

The bZIP and DNA-binding with One Finger (DOF) transcription factor families serve as critical links connecting nitrogen signaling with storage protein gene expression [142,143]. In maize, the O_2_ and PBF complex regulates α-zein and other prolamin genes [144,145]. Both expression and activity increase under elevated nitrogen supply.

Similar modules operate in rice (RISBZ1/bZIP58–RPBF) and wheat (SPA–WPBF) [146,147]. These transcription factors and their cognate cis-regulatory elements (GCN4-like motifs, P-box and O_2_-box) collectively constitute the core “nitrogen-responsive–endosperm-specific” regulatory unit [148].

The temporal activity of this regulatory module aligns closely with the post-heading/post-anthesis window targeted by late-stage nitrogen application. The responsiveness of bZIP–DOF complexes to nitrogen supply during the 5–20 DAA period means that late nitrogen application can directly enhance the transcriptional output of storage protein genes during their peak expression window, providing a mechanistic basis for the observed increases in grain protein content and gluten quality parameters.

#### 4.2.3. NAC/WRKY/MYB Factors Governing Senescence and Remobilization

The NAC (NAM, ATAF1/2, and CUC2), WRKY (WRKY transcription factor), and MYB (myeloblastosis-related transcription factor) transcription factor families play central roles in regulating leaf senescence and nitrogen remobilization [149,150,151]. Among these, *TaNAM-B1* (also known as *Gpc-B1*) in wheat accelerates flag leaf senescence and promotes nitrogen redistribution to developing grains, enhancing grain protein content. However, this enhancement comes at the cost of shortened grain-filling duration [152].

Most modern high-yielding wheat varieties carry loss-of-function alleles of *TaNAM-B1*, exemplifying the NAC-mediated trade-off between yield and protein content [153]. This trade-off has direct implications for late-stage nitrogen management: in varieties with functional NAM-B1, late nitrogen application may partially compensate for the shortened filling duration by sustaining carbon supply, while in varieties with non-functional alleles, the same application predominantly enhances protein accumulation without the penalty of premature senescence. Understanding the NAM-B1 allelic status of target varieties is therefore important for predicting the quality outcomes of late nitrogen application. In rice and maize, related NAC transcription factors including *OsNAC5/9/10* and *ZmNAC34* participate in stress responses and endosperm development [154,155]. WRKY and MYB factors interact with NAC and NLP genes, jointly determining senescence, timing and nitrogen remobilization, and the progression of grain filling [156,157]. The key nitrogen-responsive transcription factors discussed above are summarized in Table 1.

### 4.3. Crosstalk Between Hormone and Nitrogen Signaling

Nitrogen signals do not modulate individual hormonal pathways in isolation but rather reprogram the overall hormonal landscape during grain filling, creating an interconnected regulatory network that coordinately governs source–sink dynamics [159]. Understanding this network requires examining how nitrogen simultaneously reshapes hormonal homeostasis and how the resulting hormone–hormone interactions collectively determine grain quality outcomes.

#### 4.3.1. Nitrogen-Mediated Reprogramming of Hormonal Homeostasis

Nitrogen availability concurrently modulates the biosynthesis and catabolism of multiple hormones. High-nitrogen conditions upregulate CK biosynthetic genes (*OsIPT4* and *OsIPT5*) while late-stage nitrogen application suppresses CK degradation by inhibiting *OsCKX2* expression, collectively elevating endogenous CK levels [160,161,162]. Simultaneously, elevated nitrogen supply enhances ABA accumulation in developing grains [163], modifies IAA concentrations in developing spikes through YUCCA-mediated biosynthesis [164,165], and upregulates *GA2-oxidase* (*GA2ox*) genes to reduce bioactive GA levels [166]. Ethylene biosynthesis is stimulated during nitrogen remobilization from senescing tissues [167], while strigolactone production responds inversely to nitrogen availability [168]. These concurrent hormonal shifts establish a new multi-hormonal equilibrium whose integrated output—rather than any single hormonal change—determines grain development outcomes.

#### 4.3.2. Coordinated Regulation of Source Activity

At the source end, the CK–ETH antagonism serves as the primary axis controlling functional leaf duration. CK-mediated maintenance of chloroplast integrity and Rubisco activity delays senescence and extends carbon assimilation, while type-B ARR transcription factors directly activate nitrogen metabolism genes, establishing a CK–nitrogen positive feedback loop [169,170]. Opposing this, ethylene promotes nitrogen remobilization from vegetative tissues at moderate concentrations but triggers premature senescence and early termination of grain filling at excessive levels [167]. CK and SL further engage in crosstalk that functions as a nitrogen-status-dependent switch: under nitrogen sufficiency, elevated CK suppresses SL signaling, directing assimilates toward reproductive organs; under deficiency, enhanced SL promotes root-based nitrogen foraging [168]. Late-stage nitrogen application leverages this network by simultaneously boosting CK, moderating ETH, and attenuating SL, thereby extending the functional window of source organs [162].

#### 4.3.3. Integrated Control of Sink Development and Storage Accumulation

At the sink end, IAA drives endosperm cell division and sink capacity establishment through the YUCCA–ARF–PIN module, while nitrogen-induced GA inactivation via GA2ox redirects assimilates from vegetative organs toward developing grains [164,166]. As grain filling progresses, ABA assumes a central role: nitrogen-enhanced ABA activates the PYR/PYL–PP2C–SnRK2 cascade, with downstream bZIP transcription factors (e.g., ABI5) promoting storage protein and LEA protein gene expression [171,172]. Critically, this ABA-regulated sink biosynthesis is functionally coupled to CK-regulated source activity—CK sustains the nitrogen supply that provides amino acid substrates for ABA-induced storage protein accumulation, illustrating how the hormonal network integrates across the source–sink axis to determine final grain protein content.

#### 4.3.4. Implications for Nitrogen Management

The ABA/GA ratio in developing grains serves as a key quality-determining balance: late-stage nitrogen application synergistically strengthens ABA signaling while suppressing GA levels, promoting storage compound accumulation. However, excessive nitrogen can over-amplify ABA, disrupt the ABA/GA equilibrium, and compromise normal grain-filling progression [173]. This network perspective explains the well-documented dose-dependency of late-stage nitrogen effects—moderate application optimizes the multi-hormonal balance, whereas excessive application perturbs it—and underscores the idea that precision nitrogen management must target the holistic hormonal network response rather than individual pathways.

### 4.4. Coordination of Carbon–Nitrogen Metabolism and Storage Compound Accumulation

#### 4.4.1. TOR–SnRK1 Balance as a Metabolic Integrator

The antagonistic target of rapamycin (TOR) and sucrose non-fermenting 1-related kinase 1 (SnRK1) signaling pathways serve as central integrators of cellular carbon and nitrogen status [174].

TOR promotes anabolic processes and is activated under high energy and nitrogen supply [175]. Conversely, SnRK1 is activated under carbon limitation [176]. The GCN2 kinase senses amino acid deficiency and adjusts protein translation rates [177].

For the purposes of this review, the key point is that appropriate late-stage nitrogen application, combined with adequate carbon supply from sustained photosynthesis, can suppress SnRK1 and GCN2 and elevate TOR activity, thereby facilitating coordinated starch and protein. However, excessive late nitrogen may paradoxically inhibit grain filling—a phenomenon termed “high nitrogen inhibition of grain filling”—potentially through disruption of the TOR–SnRK1 balance when carbon supply becomes limiting relative to the elevated nitrogen input [176]. This framework also helps reconcile conflicting field observations, as the TOR–SnRK1 set-point during grain filling is highly sensitive to concurrent carbon supply (radiation/temperature) and thus determines whether late-stage nitrogen enhances storage deposition or triggers carbon limitation-induced filling inhibition. This metabolic framework explains the nonlinear dose–response relationships observed in field studies (Section 3.2.3) and provides a molecular rationale for the crop-specific dosage thresholds discussed in Section 3.3.3.

#### 4.4.2. Nitrogen Assimilation and Amino Acid Transport to Grains

The GS–GOGAT cycle constitutes the core pathway for primary nitrogen assimilation [101]. Cytosolic GS1 primarily participates in nitrogen remobilization from senescing tissues, while plastidic GS2 contributes to primary assimilation [178]. *OsGS1;1* and *TaGS2* significantly affect grain nitrogen supply and protein content [179,180].

Asparagine, with its high nitrogen-to-carbon ratio, serves as a principal organic nitrogen form for phloem transport, and expression of Asparagine Synthetase 1 (ASN1) is positively responsive to nitrogen supply [181].

The AAP family plays a crucial rate-limiting role in amino acid loading and unloading [182]. *OsAAP1* in rice and *TaAAP6-3B* in wheat significantly increase grain protein content when overexpressed, demonstrating their rate-limiting role in amino acid supply to developing grains [183,184]. These transporters represent critical control points through which late-stage nitrogen application influences the quantity of nitrogen reaching developing grains. Their expression levels and transport activities during the post-heading period likely determine the marginal effectiveness of additional nitrogen application—a hypothesis that merits direct experimental testing across different cereal species and genotypes. Members of the General Amino Acid Transporter (GAT) and Lysine Histidine Transporter (LHT) families also contribute to regulating grain nitrogen supply during the filling period [185].

#### 4.4.3. Sucrose–T6P Signaling Integration

Invertase (INV) and CWIN activities determine the capacity for sucrose transport and unloading to developing grains during the filling period [186]. Nitrogen availability influences CWIN activity, thereby altering the carbon source availability and sucrose/trehalose-6-phosphate (T6P) levels accessible to the grain and indirectly regulating the partitioning between starch and protein biosynthesis [187].

T6P, a signaling molecule reflecting sucrose availability, promotes anabolic metabolism by inhibiting SnRK1 [188]. The T6P–SnRK1–TOR regulatory axis functions as a critical integrative hub where carbon and nitrogen signals converge to coordinate starch and protein biosynthesis rates [189]. Under late-stage nitrogen application, the interplay between elevated amino acid supply (stimulating protein synthesis) and sustained sucrose supply (maintaining T6P levels and TOR activity) determines the final carbon-to-nitrogen ratio of mature grains. When both inputs are balanced—achievable through appropriate nitrogen dosage combined with maintained photosynthetic capacity—coordinated enhancement of both starch and protein content is possible. This integrated signaling framework provides the molecular basis for the “optimal dosage window” concept that is central to effective late-stage nitrogen management.

### 4.5. Spatiotemporal Expression of Storage Protein and Starch Synthesis Genes

#### 4.5.1. Storage Protein Gene Expression Patterns

Storage protein genes exhibit endosperm-specific expression patterns aligned with late-stage nitrogen application windows that are temporally aligned with late nitrogen application windows, making their regulation directly relevant to the quality effects discussed in this review. Storage protein gene promoters in wheat contain GCN4-like, P-box, and O_2_-box elements [190]. Expression peaks at 10–20 DAA and responds strongly to post-anthesis nitrogen, forming the molecular basis for enhancing protein content and gluten quality [191]. Rice GluA/GluB and prolamin genes increase expression rapidly at 5–20 DAA, are regulated by RISBZ1–RPBF, and are responsive to nitrogen supply changes [192]. Among maize zein family members, α-zein is most nitrogen-responsive, with expression initiating 10 days after pollination [145]. In barley, hordein gene promoters contain GCN4-like and P-box elements, and under BPBF and BLZ1/BLZ2 regulation, hordein transcription increases rapidly at 10–25 DAA with high nitrogen sensitivity [193,194].

The convergence of peak storage protein gene expression with the typical timing of late-stage nitrogen application (heading to 10–15 DAA) explains why this management practice is particularly effective at modulating grain protein content. The nitrogen-responsive cis-elements in storage protein gene promoters provide direct transcriptional targets for the NLP and bZIP–DOF regulatory modules discussed in Section 4.2, establishing a clear molecular pathway from late nitrogen application to grain protein accumulation.

#### 4.5.2. Starch Synthesis Gene Regulation

Starch biosynthesis requires coordinated action of multiple enzymes, including AGPase, GBSS, SS, SBE, and DBE. Peak expression of genes encoding these enzymes predominantly occurs at 20-30 DAA—generally later than storage protein genes [40,44,46]. Expression is primarily regulated by carbon source availability (sucrose/T6P levels) and developmental signals rather than by nitrogen directly [40,44,46]. Nevertheless, nitrogen exerts decisive indirect effects on starch content and amylose-to-amylopectin ratio by altering the carbon-to-nitrogen ratio, modulating the TOR–SnRK1–T6P pathway, and influencing source–sink relationships [189]. Recently, TaPIL1 was identified as a transcription factor that specifically activates key starch synthesis genes (AGPS1a, GBSSI, and BEIIb) [195]. In barley, HvAGP and HvGBSS1a expressions during mid-to-late grain filling determine final starch content, gelatinization properties, and β-glucan content [196,197]. Appropriate late-stage nitrogen application can help maintain carbon supply while improving nitrogen use efficiency, thereby achieving coordinated enhancement of both starch and protein content [198].

### 4.6. Epigenetic Regulation and Non-Coding RNA

#### 4.6.1. Histone Modifications in Nitrogen and Developmental Regulation

Histone modifications play vital roles in regulating nitrogen-responsive and grain development genes. Activating modifications including H3K4me3 (Histone H3 Lysine 4 trimethylation) and H3K36me3 (Histone H3 Lysine 36 trimethylation) are associated with transcriptional activation of nitrogen genes [199]. SDG8 (Set Domain Group 8), catalyzing H3K36me3 deposition, affects both nitrogen use efficiency and grain yield [200]. The repressive modification H3K27me3 (Histone H3 Lysine 27 trimethylation) and its demethylase JMJ705 are implicated in the temporal regulation of storage protein gene expression [201,202]. The histone acetyltransferase GCN5 and deacetylases HDA19 and OsHDA710 play important roles in nitrogen responses, senescence regulation, and storage substance accumulation [203,204,205]. Whether late-stage nitrogen application modulates the histone modification landscape at storage protein and starch synthesis gene loci during grain filling remains an important but largely unexplored question.

#### 4.6.2. DNA Methylation Dynamics

Nitrogen treatment induces both demethylation and remethylation events at nitrogen metabolism gene promoters [206]. In wheat, promoter methylation levels of storage protein genes exhibit negative correlation with their expression levels, suggesting that late-stage nitrogen application may regulate protein accumulation partially through effects on DNA methylation status [207]. Elucidating the specific mechanisms underlying nitrogen-induced methylation changes at grain quality gene loci during the post-anthesis period represents an important frontier for future research.

#### 4.6.3. MicroRNA-Mediated Regulation

MicroRNAs (miRNAs) function as post-transcriptional regulators of nitrogen response pathways [208]. The miR167–ARF and miR169–NF-YA modules regulate nitrogen-responsive processes [209,210]. The miR444–MADS participates in nitrogen response regulation [211]. The miR156–SPL and miR396–GRF modules regulate grain size and storage substance accumulation [212,213]. These miRNA circuits provide fine-tuning layers integrating nitrogen signals with developmental programs during grain filling.

Long non-coding RNAs (lncRNAs) and circular RNAs (circRNAs) exhibit significant spatiotemporal expression during grain development [214,215]. Although specific roles of individual lncRNAs and circRNAs in mediating late-stage nitrogen effects on grain quality remain largely unexplored, emerging evidence suggests that they may participate in the epigenetic regulation of nitrogen-responsive gene networks during grain filling, representing a promising frontier for future investigation.

Section 4 highlights multi-layer molecular networks that connect nitrogen signaling with hormone crosstalk, metabolic regulation, and storage compound biosynthesis during grain filling. Building on this mechanistic framework, Section 5 discusses key challenges and practical optimization strategies for precision late-stage nitrogen management.

## 5. Current Challenges and Optimization Strategies

### 5.1. Optimization of Nitrogen Application Timing and Developmental Stage Matching

#### 5.1.1. Critical Developmental Windows for Nitrogen Application

The effectiveness of late-stage nitrogen application is largely contingent upon precise alignment between application timing and specific crop developmental stages [216]. The developmental windows described below primarily refer to wheat and rice under typical temperate and subtropical production systems, whereas barley generally exhibits a relatively shorter grain-filling duration and a narrower response window. Different developmental phases present distinct opportunities for quality manipulation through nitrogen management, and understanding these windows is essential for optimizing application strategies.

In wheat and rice, nitrogen application during the period between 5 and 7 days before anthesis can promote endosperm cell proliferation and increase the number of starch granule initiation sites, thereby establishing the physical foundation for subsequent grain filling [217]. Application at the heading stage effectively maintains the photosynthetic capacity of functional leaves and promotes nitrogen accumulation during early grain development [218]. Nitrogen applied precisely at the anthesis stage represents the most timely intervention, directly meeting the nitrogen demands of grain development and exhibiting the most pronounced effects on protein content enhancement [219]. The grain-filling initiation stage (7–14 DAA) marks the commencement of active starch and protein biosynthesis. Nitrogen application during this window enhances sink activity and increases the grain filling [220]. The rapid grain-filling stage (14–28 DAA) represents the period of maximum dry matter accumulation, characterized by high nitrogen demand. Late-stage nitrogen application during this phase maintains source organ functionality and ensures sustained assimilate supply to developing grains [221].

#### 5.1.2. Real-Time Diagnosis and Precision Application Technologies

In recent years, real-time nitrogen application technologies based on crop nutrient diagnosis have made precision late-stage nitrogen management increasingly feasible [222]. Vegetation indices derived from canopy spectral reflectance, including the Normalized Difference Vegetation Index (NDVI) and Red Edge Normalized Difference Vegetation Index (NDRE), provide reliable and non-destructive indicators of crop nitrogen nutritional status [223]. Integration of these spectral diagnostic approaches with Unmanned Aerial Vehicle (UAV) or satellite remote sensing platforms enables large-scale crop nitrogen assessment and variable-rate fertilization decision-making across entire production fields [224,225].

Although the calibration of spectral indices for late-stage nitrogen diagnosis remains challenging, as relationships between spectral signatures and nitrogen status may differ substantially during reproductive development compared to vegetative growth phases, the development of crop-specific and growth stage-specific calibration protocols could be a solution for optimizing the accuracy of real-time nitrogen recommendations.

### 5.2. Nitrogen Application Dosage Determination and Quality-Oriented Management

#### 5.2.1. Crop-Specific Requirements Based on Quality Objectives

The determination of appropriate late-stage nitrogen application dosage requires comprehensive consideration of cultivar characteristics, target quality specifications, and end-use requirements. Those crops targeting high protein content—such as bread wheat and feed maize—require relatively higher late-stage nitrogen inputs, with the upper limit of application primarily constrained by risks of lodging, excessive vegetative growth, delayed maturity, and adverse environmental impacts rather than quality considerations per se. For crops requiring protein content within defined moderate ranges, like malting barley, precise control of late-stage nitrogen application is required to maintain protein content within specifications acceptable for brewing applications. Crops targeting relatively low protein content—such as premium japonica rice—require careful limitation of late-stage nitrogen to prevent excessive protein accumulation that would compromise eating quality and simultaneously ensure adequate yield performance.

#### 5.2.2. Diminishing Marginal Returns and Threshold Effect

Late-stage nitrogen application exhibits characteristic diminishing marginal returns for grain quality improvement [226]. As application rates increase, the incremental gain in protein content progressively decreases, while the risk of negative effects—including reduced starch content, decreased grain test weight, and compromised processing quality—gradually escalates [227]. Studies indicate that within the range of 0–60 kg N/ha, protein content increases relatively substantially in response to late-stage nitrogen application. Beyond 60 kg N/ha, the rate of increase diminishes noticeably. Above 100 kg N/ha, negative effects may begin to outweigh benefits [228]. These threshold values vary considerably among crops, cultivars, and environmental conditions, underscoring the need for site-specific calibration of optimal application rates based on local validation trials.

### 5.3. Nitrogen Form Selection and Use Efficiency Optimization

#### 5.3.1. Comparative Characteristics of Nitrogen Forms

The chemical form of applied nitrogen fertilizer significantly influences crop absorption efficiency and quality regulation outcomes [229]. The principal nitrogen forms utilized for late-stage application include nitrate nitrogen, ammonium nitrogen, and amide nitrogen (urea), each presenting distinct advantages and limitations [230].

Nitrate nitrogen (NO_3_^−^) exhibits high mobility in soil solution and minimal fixation by soil colloids, enabling rapid movement to the root absorption zone [231]. However, nitrate uptake requires active transport mechanisms that consume metabolic energy (ATP), and assimilation necessitates sequential reduction by nitrate reductase (NR) and nitrite reductase (NiR) before incorporation into amino acids [232].

Ammonium nitrogen (NH_4_^+^) is readily adsorbed by soil colloids, resulting in limited mobility but effective retention in the rhizosphere zone [233]. As a direct substrate for the GS-GOGAT assimilation pathway, ammonium nitrogen is metabolized with high efficiency. However, elevated ammonium concentrations may exert phytotoxic effects, particularly in sensitive crop species or under conditions limiting ammonium assimilation [234].

Urea (amide nitrogen) is the most widely utilized nitrogen fertilizer form, characterized by high nitrogen content (46%) that facilitates transport and application logistics. Following soil application, urea undergoes urease-mediated hydrolysis to ammonium nitrogen. However, this conversion process may result in substantial ammonia volatilization losses, particularly under conditions of elevated temperature and alkaline soil pH [235].

#### 5.3.2. Controlled-Release Fertilizers and Enhanced Efficiency Technologies

Controlled-release nitrogen fertilizers are an important technological advancement for optimizing nitrogen supply dynamics [236]. These products delay nitrogen release through physical polymer coatings or chemical inhibitors, enabling improved temporal matching between nitrogen availability and crop demand patterns.

For late-stage nitrogen applications, controlled-release formulations offer several potential advantages, including extended duration of nitrogen supply, improved nitrogen use efficiency, and more stable supply intensity throughout the grain-filling period [236]. However, the release characteristics of these products are substantially influenced by environmental variables including temperature and soil moisture content [237]. Systematic characterization of release kinetics under varying climatic conditions is required to enable reliable predictions of nitrogen availability and inform appropriate product selection.

### 5.4. Environmental Modulation of Nitrogen Application Effectiveness

#### 5.4.1. Temperature, Radiation, and Precipitation Effects

Temperature exerts profound effects on late-stage nitrogen application outcomes through the regulation of both crop metabolic activity and soil nitrogen transformation processes [238]. Under optimal temperature conditions, the protein-enhancing effects of late-stage nitrogen application are most pronounced. However, elevated temperatures accelerate leaf senescence, effectively shortening the window for nitrogen uptake and assimilation and potentially negating the intended benefits of late application [239]. The increasing frequency of heat stress events under climate change scenarios has imposed a growing challenge for reliable late-stage nitrogen management.

Solar radiation is another critical environmental factor modulating the effectiveness of late-stage nitrogen application. Light intensity directly governs photosynthetic carbon fixation capacity, which provides the energy and carbon skeletons essential for nitrogen assimilation into amino acids and proteins [240]. Under high-radiation environments, late-stage nitrogen application more effectively enhances grain protein content because sufficient photosynthate supply supports the energy-intensive processes of nitrate reduction, amino acid biosynthesis, and protein assembly in developing grains [241]. Conversely, prolonged low-light conditions—such as those caused by cloudy weather or high planting density—limit photosynthetic output, reduce the availability of reductants (NADH and ferredoxin) required for nitrogen assimilation, and consequently diminish the quality-enhancing benefits of applied nitrogen [242]. Furthermore, light quality and photoperiod influence the expression of key nitrogen metabolism genes, including nitrate reductase (NR) and glutamine synthetase (GS), whose activities exhibit diurnal regulation patterns closely coupled to photosynthetic electron transport [243]. The interaction between radiation and nitrogen is particularly relevant during grain filling, as the simultaneous demand for carbon assimilates (for starch synthesis) and nitrogen assimilates (for storage protein accumulation) creates a metabolic competition that is ultimately constrained by photosynthetic capacity [244]. Consequently, the effectiveness of late-stage nitrogen application is inherently dependent on the prevailing radiation environment, and nitrogen management strategies should account for regional and seasonal light availability to maximize quality outcomes.

Precipitation patterns critically influence the fate of applied nitrogen fertilizers. Moderate rainfall promotes nitrogen dissolution and facilitates root uptake. However, excessive precipitation leads to nitrogen losses through leaching below the root zone and surface runoff, particularly on sloping terrain [245]. Studies have demonstrated that continuous rainfall events following nitrogen application can reduce nitrogen use efficiency by 30–50%, severely compromising expected quality enhancement outcomes [246]. The inherent unpredictability of precipitation during reproductive growth phases represents a substantial source of uncertainty in late-stage nitrogen management.

#### 5.4.2. Soil Properties and Nitrogen Dynamics

Soil physical and chemical properties directly influence nitrogen availability and crop uptake efficiency. Clay-textured soils with high cation exchange capacity exhibit strong nutrient retention, resulting in more prolonged effectiveness of applied nitrogen. In contrast, sandy soils with limited retention capacity may require more frequent applications at reduced rates to maintain adequate nitrogen availability while minimizing losses [247]. Soil pH influences nitrogen form transformations: alkaline conditions increase ammonia volatilization risk, while strongly acidic conditions may limit nitrification rates and potentially enhance ammonium toxicity [248]. Soil microbial communities regulate organic nitrogen mineralization and participate in various nitrogen cycling processes, thereby influencing the ultimate fate of applied fertilizer nitrogen [249]. Understanding these soil-mediated processes is essential for developing site-specific nitrogen management recommendations.

### 5.5. The Contradiction and Coordination Between High Yield and High Quality

High yield and quality form a ‘see-saw’ relationship in the competition for assimilates, and this trade-off effect is particularly evident in later nitrogen application practices [22]. Increasing nitrogen application can improve protein content, but it may affect yield by promoting vegetative growth, delaying maturity, or increasing the risk of disease [9,250]. The molecular basis of this trade-off involves NAC transcription factor-mediated regulation of senescence timing, as discussed in Section 4.2. Accelerated senescence promotes nitrogen remobilization and protein accumulation but truncates grain-filling duration, potentially limiting yield [251]. Conversely, delayed senescence extends grain filling but may restrict protein accumulation. Breaking this fundamental trade-off requires either genetic modification of the underlying regulatory networks or management strategies that optimize the timing and intensity of nitrogen supply to achieve acceptable compromises between competing objectives [252].

Reconciling the yield–quality conflict requires comprehensive, multi-faceted approaches that address genetic, environmental, and management dimensions simultaneously. Variety selection constitutes a fundamental component: development and deployment of nitrogen-efficient cultivars can achieve higher protein content at equivalent nitrogen inputs or maintain quality standards under reduced nitrogen conditions, effectively shifting the trade-off frontier [253,254]. Optimization of cultivation practices is equally critical. Stage-specific nitrogen regulation strategies can ensure adequate population establishment during vegetative growth while meeting quality objectives during reproductive development. Integrated water and nitrogen management improves nitrogen use efficiency, potentially enabling reduced nitrogen inputs while maintaining quality targets [255,256]. The concept of integrated genotype × environment × management (G × E × M) optimization offers a systematic framework for addressing yield–quality conflicts. This approach recognizes that optimal management strategies are inherently context-dependent, varying with genotype characteristics, environmental conditions, and quality objectives. He et al. [257] employed the process-based APSIM-Canola model to investigate G × E × M interactions, demonstrating its capability to accurately simulate seed yields across diverse climatic zones. Agrahari et al. [258] utilized simulation approaches to examine G × E × M interactions for sustainable intensification of rainfed wheat cropping systems in Morocco. Implementation of G × E × M optimization requires robust decision support systems capable of integrating diverse data sources and providing site-specific, quality-oriented recommendations.

### 5.6. Special Challenges in Malting Barley Nitrogen Management

#### The Narrow Protein Window Constraint

Malting barley presents unique challenges for late-stage nitrogen management. As discussed earlier, the brewing industry imposes stringent grain protein content specifications within the narrow range of 9.5–11.5% [3]. This requirement demands that nitrogen management achieve a delicate balance between ensuring complete grain development through adequate nitrogen supply and avoiding excessive protein accumulation through nitrogen limitation. Compared to wheat or rice, where moderate protein over-accumulation is generally tolerable or even desirable, the margin for error in malting barley nitrogen application decisions is extremely limited, placing extraordinary demands on application precision [27,259].

These challenges are compounded by the inadequate development of barley-specific diagnostic standards and decision support systems. Existing nitrogen status evaluation indicators—including critical leaf nitrogen concentration thresholds, SPAD-based chlorophyll indices, and canopy spectral reflectance parameters—have been predominantly established and validated for wheat, and their direct application to malting barley may lead to systematic errors in fertilization recommendations [27,260]. Furthermore, most nitrogen diagnostic frameworks are calibrated against yield responses rather than quality outcomes, rendering them poorly suited for malting barley, where the primary management objective is protein content control within defined limits rather than yield maximization [261]. Development of barley-specific, quality-oriented nitrogen diagnostic tools—potentially integrating real-time canopy sensing with grain protein prediction models—represents an important priority for future research and extension efforts [262].

### 5.7. Emerging Technologies and Future Research Directions

#### 5.7.1. Smart Agriculture and Precision Nitrogen Management

The rapid advancement of smart agriculture technologies has opened new avenues for precision nitrogen management [263]. UAV-based remote sensing enables rapid acquisition of spectral information across large production areas, with vegetation indices providing an indirect but reliable assessment of crop nitrogen status [224,225]. Integration of these sensing capabilities with artificial intelligence (AI) and machine learning algorithms enables the development of data-driven fertilization decision models that can generate intelligent recommendations for nitrogen application timing and rates based on real-time field conditions [253].

The coupling of crop growth simulation models with nitrogen management decision systems provides powerful theoretical tools for precision fertilization [255]. Established crop models such as Decision Support System for Agro-Technology (DSSAT) and APSIM have been successfully applied to simulate yield and quality responses under diverse nitrogen management scenarios [257]. Integration of these mechanistic models with real-time weather data, soil information, and field monitoring systems is expected to substantially enhance the accuracy and timeliness of nitrogen management recommendations [258].

#### 5.7.2. Genetic Improvement for Nitrogen Use Efficiency

Developing elite crop cultivars with enhanced nitrogen use efficiency through marker-assisted selection or genome-editing technologies offers complementary approaches to improved nitrogen management [264]. The integration of genetically improved cultivars with precision nitrogen application technologies can produce synergistic effects exceeding those achievable through either approach alone. This integrated “superior genotype + optimal management” strategy represents a promising direction for future nitrogen management systems and offers a pathway toward simultaneously achieving productivity, quality, and sustainability objectives [265].

Under the context of global carbon neutrality goals, exploration of synergistic pathways between late-stage nitrogen application optimization and greenhouse gas emission reduction represents an emerging priority. Construction of “high-quality, high-efficiency, and low-carbon” integrated nitrogen management systems represents an important frontier for future research and practice.

## 6. Conclusions

### 6.1. Conclusion

Quality trait modifications: Late-stage nitrogen application generally exhibits positive effects on grain protein content across cereal crops, although the magnitude and desirability of those effects vary substantially depending on end-use requirements. Concurrently, starch content may decrease due to carbon–nitrogen competition effects at the metabolic level. Apart from total protein and starch content, quality attributes including storage protein composition, amino acid profiles, and starch physicochemical properties are all modulated by late-stage nitrogen availability.

Physiological mechanisms: Late-stage nitrogen application influences grain filling through multiple interconnected pathways: delaying functional leaf senescence, maintaining photosynthetic apparatus activity, and promoting assimilate translocation to developing grains. These physiological responses provide both the material foundation and temporal extension necessary for sufficient accumulation of storage compounds.

Molecular regulatory networks: Late-stage nitrogen signals are perceived through NRT or AMT families and trigger downstream response cascades mediated by core transcription factors including NLPs. Through the SnRK1–TOR–T6P regulatory axis, which coordinates carbon and nitrogen metabolism in developing grains, the integration of nitrogen status with cellular energy balance could be accomplished. Ultimately, transcriptional regulation by bZIP–DOF modules and epigenetic modifications including H3K4me3 deposition collectively determine the expression of genes encoding starch biosynthetic enzymes and storage proteins, thereby governing the accumulation patterns of storage compounds.

Crop-specific considerations: Malting barley is classified as having particularly stringent requirements for nitrogen management. Quality parameters critical for brewing—including the Kolbach index, β-glucan content, and hordein composition—are highly sensitive to late-stage nitrogen availability. The effective management window for late-stage nitrogen application is narrower than for other cereals, making precision nitrogen management particularly challenging yet critically important for this crop.

### 6.2. Current Research Limitations

Despite significant progress in understanding nitrogen regulation of grain quality, several important limitations in current knowledge warrant acknowledgment, as explained below.

Incomplete mechanistic understanding: Existing studies have focused predominantly on phenotypic observation and physiological analysis, with underlying molecular mechanisms not yet fully elucidated [266]. In particular, how nitrogen signals are integrated and converted into precise reprogramming of carbon–nitrogen metabolism—and the complete regulatory networks involved—remains incompletely characterized [267].

Limited research progress on malting barley: The late-stage nitrogen application studies on barley, particularly for malting barley, substantially fall behind those on wheat and rice. A systematic molecular atlas of nitrogen responses specific to barley is lacking. The molecular mechanisms governing the differential regulation of hordein components and β-glucan metabolism remain poorly understood [3].

Absence of high-resolution dynamic maps: High spatiotemporal resolution—“nitrogen response dynamic maps”—that capture the temporal progression of molecular and metabolic responses during grain filling is not yet available. The differential responses to nitrogen input at various stages of the grain-filling period and their molecular bases require further elucidation. The application of integrated multi-omics approaches in studies of cereal grain quality remains relatively limited, restricting systematic analysis of regulatory networks and precise identification of key control points [268].

Limited integration across scales: Current understandings of nitrogen effects on grain quality tend to be compartmentalized, with physiological, biochemical, and molecular studies often being conducted separately. Integration across these organization levels—from molecular signaling to whole-plant physiology to crop-level quality outcomes—remains a significant challenge that limits our ability to develop predictive models and targeted interventions.

### 6.3. Future Research Priorities

Based on the synthesis of current knowledge and identified limitations, several priority areas for future research can be delineated:

Development of time-series multi-omics frameworks: Future research should establish comprehensive multi-omics datasets capturing nitrogen responses across the entire grain-filling period. Through dynamic monitoring and integration of transcriptomics, metabolomics, and proteomics data, the construction of time-resolved models of nitrogen response regulatory networks becomes feasible [269]. This approach will facilitate identification of key temporal control points and molecular switches that regulate quality formation, providing a mechanistic foundation for optimizing nitrogen application timing.

Construction of G × E × M predictive models: Integration of multi-factor interaction effects encompassing G × E × M is essential for developing quality-oriented precision fertilization decision support systems [270]. By combining mechanistic crop growth models with machine learning algorithms and real-time monitoring data, intelligent recommendations for nitrogen application strategies tailored to specific ecological regions, cultivar characteristics, and quality objectives can be achieved [253].

Functional validation of key regulatory genes: Systematic functional validation of key regulatory genes using genome-editing technologies is needed to establish definitive causal relationships between specific molecular components and quality outcomes.

Climate-resilient nitrogen management: Exploration of synergistic pathways between late-stage nitrogen optimization and climate change adaptation remains an emerging priority. By integrating grain quality regulation with nitrogen environmental stewardship objectives, the development of “high-quality, high-efficiency and low-carbon” integrated nitrogen management systems can be achieved [271].

Constructing ideal model crop: Malting barley, due to its unique quality requirements and the narrow acceptable protein content range, provides an ideal model system for studying optimization mechanisms of carbon–nitrogen metabolism under nitrogen-constrained conditions. Future research on malting barley should focus on several key areas: (i) uncovering the precise regulatory mechanisms governing protein synthesis during barley grain development and the coordinated regulatory networks linking protein accumulation with starch biosynthesis; (ii) conducting systematic multi-omics studies of nitrogen responses specific to malting barley to construct crop-specific nitrogen response atlases.

## Figures and Tables

**Table 1:** Summary of nitrogen-responsive transcription factors regulating grain quality in cereals.

TF Family	Gene Name	Crop	Primary Function	Target Genes/Cis-Elements	References
NLP	*OsNLP1*	Rice	Nitrate signal transduction; activation of N uptake and assimilation genes	NRT, AMT, GS/NRE	[139]
	*OsNLP4*	Rice	Nitrate signal transduction; improvement in NUE	NRT, AMT, GS/NRE	[137]
bZIP	O_2_ (Opaque2)	Maize	Master regulator of storage protein gene expression	α-zein, prolamin genes/O_2_-box, GCN4 motif	[141,158]
	RISBZ1 (bZIP58)	Rice	Regulation of glutelin gene expression	Glutelin genes/GCN4 motif	[143]
	SPA	Wheat	Regulation of prolamin gene expression	Prolamin genes/GCN4 motif	[144]
DOF	PBF	Maize	Co-regulation of storage protein genes with O_2_	α-zein genes/P-box	[142,158]
	RPBF	Rice	Co-regulation of glutelin genes with RISBZ1	Glutelin genes/P-box	[143]
	WPBF	Wheat	Co-regulation of prolamin genes with SPA	Prolamin genes/P-box	[144]
NAC	TaNAM-B1 (Gpc-B1)	Wheat	Acceleration of senescence; promotion of N, Zn, and Fe remobilization to grains	Nutrient transporters	[152]
	*OsNAC5/9/10*	Rice	Stress response; root development enhancement	Stress-responsive genes	[151]
	*ZmNAC34*	Maize	Endosperm developmental regulation	Developmental genes	[155]
WRKY	Related members	Rice, Wheat	Regulation of senescence timing; interaction with NAC/NLP factors	Senescence-associated genes	[153]
MYB	Related members	Rice, Maize	Coordination of N remobilization and grain filling	N metabolism genes	[154]

Abbreviations: NLP, NIN-like protein; bZIP, basic leucine zipper; DOF, DNA-binding with One Finger; NRT, nitrate transporter; AMT, ammonium transporter; GS, glutamine synthetase; NUE, nitrogen use efficiency; NRE, nitrate-responsive cis-element; NAC, (NAM, ATAF1/2, and CUC2) transcription factor; WRKY, WRKY transcription factor; MYB, myeloblastosis-related transcription factor).

## Data Availability

No new data were created or analyzed in this study.
